# Storage Effects on Sample Integrity of Environmental Surface Sampling Specimens with *Bacillus anthracis* Spores

**DOI:** 10.4172/2167-0331.S1-002

**Published:** 2013-01-17

**Authors:** K. Allison Perry, Heather A. O’Connell, Laura J. Rose, Judith A. Noble-Wang, Matthew J. Arduino

**Affiliations:** Division of Healthcare Quality Promotion, National Center for Emerging and Zoonotic Infectious Diseases, Centers for Disease Control and Prevention, Atlanta, GA, USA

**Keywords:** Shipping integrity, Swab, Environmental sampling, *Bacillus anthracis*, qPCR

## Abstract

The effect of packaging, shipping temperatures and storage times on recovery of *Bacillus anthracis*. Sterne spores from swabs was investigated. Macrofoam swabs were pre-moistened, inoculated with *Bacillus anthracis* spores, and packaged in primary containment or secondary containment before storage at −15°C, 5°C, 21°C, or 35°C for 0–7 days. Swabs were processed according to validated Centers for Disease Control/Laboratory Response Network culture protocols, and the percent recovery relative to a reference sample (T_0_) was determined for each variable. No differences were observed in recovery between swabs held at −15° and 5°C, (p ≥ 0.23). These two temperatures provided significantly better recovery than swabs held at 21°C or 35°C (all 7 days pooled, p ≤ 0.04). The percent recovery at 5°C was not significantly different if processed on days 1, 2 or 4, but was significantly lower on day 7 (day 2 vs. 7, 5°C, 10^2^, p=0.03). Secondary containment provided significantly better percent recovery than primary containment, regardless of storage time (5°C data, p ≤ 0.008). The integrity of environmental swab samples containing *Bacillus anthracis* spores shipped in secondary containment was maintained when stored at −15°C or 5°C and processed within 4 days to yield the optimum percent recovery of spores.

## Introduction

During the Anthrax response in 2001, environmental surface samples (swabs, wipes, HEPA vacuum socks, etc.) were collected and shipped to laboratories for extraction and analysis [1–3]. Shipping and storage conditions such as temperature, shipping time, and sample storage were not monitored or standardized. Temperature fluctuations may have occurred and influenced laboratory results. Though *Bacillus anthracis* (BA) spores are known to be hardy, questions arose about the possibility that spores would germinate and allow for cell growth in the presence of dust and wetting agent. Another concern centered on temperature fluctuations that may occur during shipping and how this might impact the viability of spores before arriving at the laboratory [4]. Congressional reports from the Government Accountability Office [4,5] determined that answering these questions was critical in order to provide better confidence in the results of environmental sampling after an anthrax contamination event.

Several studies have investigated sampling methods for BA spores or their surrogates on various surfaces [6–10]. These studies focused on the collection and processing of samples, but did not include the important factors of storage and transportation conditions. Some investigations have looked at the influence of storage time and temperature on organisms of clinical relevance [11–13]. At present, however, no investigations have been conducted to determine the optimum storage and transportation conditions for environmental samples that may contain BA spores.

The Centers for Disease Control and Prevention/National Institute for Occupational Safety and Health (CDC/NIOSH) developed standardized sampling and shipping methods for BA spores, currently in use by first responders [14]. A method for processing BA swab samples in the laboratory was developed, optimized, validated, and is currently in use by Laboratory Response Network (LRN) laboratories [15–17]. The purpose of this study was to provide the data for the bridging step between the sampling collection and the laboratory processing method, an essential part of providing a validated, standardized method for the investigation of a bioterrorism event. In this study, we investigated the effects of storage conditions such as temperature, time, containment, and presence of dust on recovery of viable BA spores from macrofoam swabs.

## Materials and Methods

### Spore preparation

Spores of *Bacillus anthracis* Sterne 34F2 (Colorado Serum, Denver, CO) were prepared as described previously [15]. The titer of the working stocks was checked by dilution and plating on Trypticase™ Soy Agar with 5% Sheep Blood (TSAB, Beckton, Dickinson and Co, Sparks, MD). Colony forming units (CFU/ml) were counted after incubation at 35°C for 18–24 h.

### Swab inoculation, storage, and processing

Macrofoam swabs (Puritan Medical, Guilford, ME) (n=10) were pre-moistened with 300 µl phosphate buffered saline with 0.02% Tween^®^ 80 (PBST) or neutralizing buffer (NB, Beckton, Dickinson and Co, Sparks, MD) and then placed into 15-cc polypropylene centrifuge tubes. The swabs were then inoculated with approximately 50 µl of the working stock spore suspension for a total of 350 µL of fluid on the swab head. The quantity of spore suspension was adjusted to meet the final colony forming units (CFU) swab targets of 10^4^, or 10^2^ CFU/swab. Ten swabs were inoculated for each temperature, storage time, and containment parameter being tested. Each set of ten 15-cc tubes were sealed with Parafilm^®^ (Pechiney Plastic Packaging Company, Chicago, IL) and placed in one diagnostic specimen transport bag (Air Sea Atlanta, Atlanta, GA), considered primary containment (PC). Swabs with dust added (see below) were also evaluated after storage in secondary containment (SC), which consisted of placing the specimen transport bags inside a 10 L tinplate drum (Air Sea Atlanta, Atlanta, GA). The 10 L tinplate drum, which meets the requirements specified in CFR 173.196 [18] was chosen in order to hold the large number of swabs needed for each experiment. The containers were stored at the following temperatures −15°C, 5°C, 21°C, or 35°C (± 2°C). The swabs were removed after storage for 0, 1, 2, 4, and 7 days, and processed according to the validated LRN protocols [15]. The reference sample (T_0_) was processed 1 hour after inoculation. Five mL of PBST was added to the tubes containing the swabs, and the tubes were alternately vortexed and sonicated for 30 seconds, the cycle being repeated three times. The swabs were aseptically removed, the eluent diluted in series, and 100 µl of each dilution plated on TSAB in triplicate. For swabs inoculated with 10^2^ CFU/swab, 1 mL of the eluent was vacuum filtered onto gridded 0.45 µm pore size mixed cellulose ester filters (Microfunnel; Pall Corporation, Port Washington, NY) in triplicate and the filters were aseptically placed on TSAB. All plates were incubated overnight at 35°C, and colonies with characteristic BA colony morphology were counted.

To simulate dirty environmental samples, swabs were pre-moistened with NB containing 10 mg/mL of Arizona Test Dust (ATD, A-3 Powder Tech, Inc, Minneapolis, MN). The ATD was previously cultured and found to have an intrinsic consortium of *Bacillus* spp., actinomycetes, and fungi [19]. Swabs without ATD were evaluated in PC only.

### qPCR

Representative BA colonies were confirmed by the CDC/LRN qPCR protocol [20] though the pX02 primer and probe set were not employed since BA Sterne is lacking this plasmid. The Light Cycler Fast-Start DNA Master Hybridization probes were used (Roche Molecular Biochemical Inc., Indianapolis, IN) and analysis was performed with a 7500 Fast real time PCR system (Applied Biosystems, Foster City, CA).

### Data analysis

The percent recovery (%R) at each sampling time relative to the reference T_0_ CFU/ml was calculated. The mean %R, for each temperature and containment for each day (1, 2, 4, and 7) was determined. The data for each parameter was pooled upto days 1, 2, 4 and 7 for comparisons to improve statistical strength. The standard deviation and coefficient of variation (CV) of the pooled %R data were determined for each study parameter. Analysis of variance (ANOVA) and graphs were generated with PASW^®^ software, version 18 (IBM, Armonk, NY) with significant values designated as p<0.05. The mean percent recovery (%R) and 95% confidence intervals (CI) were plotted across all 7 days for all 4 temperatures and inoculum levels. The CFU/ml was also log_10_ transformed and the change relative to T_0_ was determined for each storage variable. The sensitivity for detection of BA was reported as the percent of presumptive colonies tested that were confirmed by qPCR.

## Results

No significant difference in recovery was seen between swabs pre-moistened with PBST and those pre-moistened with NB for all inoculum levels when the storage data up to 2 days for all temperatures was pooled (p=0.76). Since NB is easily obtained from commercial sources, and because it would be preferred for post-decontamination sampling, the remainder of the study was conducted with NB as the pre-moistening agent.

The %R of swabs with and without ATD was compared when stored at 5°C in primary containment (PC). The pooled data for swabs stored for 2 days and 7 days showed that swabs without ATD demonstrated a greater %R than swabs with ATD 10^4^ CFU/swab inoculum (p ≤ 0.001). However, swabs with ATD at 10^2^ CFU/swab inoculum had greater %R (p=0.004) than those without ATD. Less variability in the %R (lower CV) was seen when ATD was present, regardless of the inoculum (Table 1).

Organisms present in the ATD were able to multiply when swabs were held at 35°C, resulting in culture plates with large numbers of competing organisms. This made identifying BA colonies on the plates and filters more difficult, especially at the 10^2^ CFU/swab inoculum level. Representative BA colonies were confirmed by qPCR. The sensitivity of selecting BA by colony morphology alone was found to be 100% at the 10^4^ CFU inoculum level, and 96% at the lower inoculum level (Table 2).

The %R of spores from swabs with ATD stored at 5°C was compared to −15°, 21°, and 35°C in SC (Table 3). No significant differences were observed between 5°C, −15°, and 21°C when the pooled data for storage up to 2 days was analyzed, regardless of inoculum level (p ≥ 0.09). Storage at 35°C demonstrated a significantly lower %R (p ≤ 0.01) than those stored at 5°C. When data was pooled for storage up to 7 days, swabs held at −15° and 5°C provided significantly better %R relative to T_0_ than if held at the higher temperatures, 21°C (p ≤ 0.02) or 35°C (p ≤ 0.04). When swabs were held at 5°C, no significant differences were seen in %R if processed at 1, 2, or 4 days. However, swabs at the 10^2^ inoculum that were processed on day 7, had significantly lower %R than if processed at day 2 (p=0.03). The cumulative %R of swabs stored at 5°C for up to 2 days was 102.7% and 102.5% at the 10^4^ and 10^2^ inoculum levels, respectively. If stored at 21°C, the %R either increased significantly (10^2^ inoculum) or decreased significantly (10^4^ inoculum). If stored at 35°C, a significant decline in %R of spores was noticed, regardless of inoculum level (p ≤ 0.040). A compilation of all the p-values for temperature comparisons can be seen in table 4.

**Figure 1:**
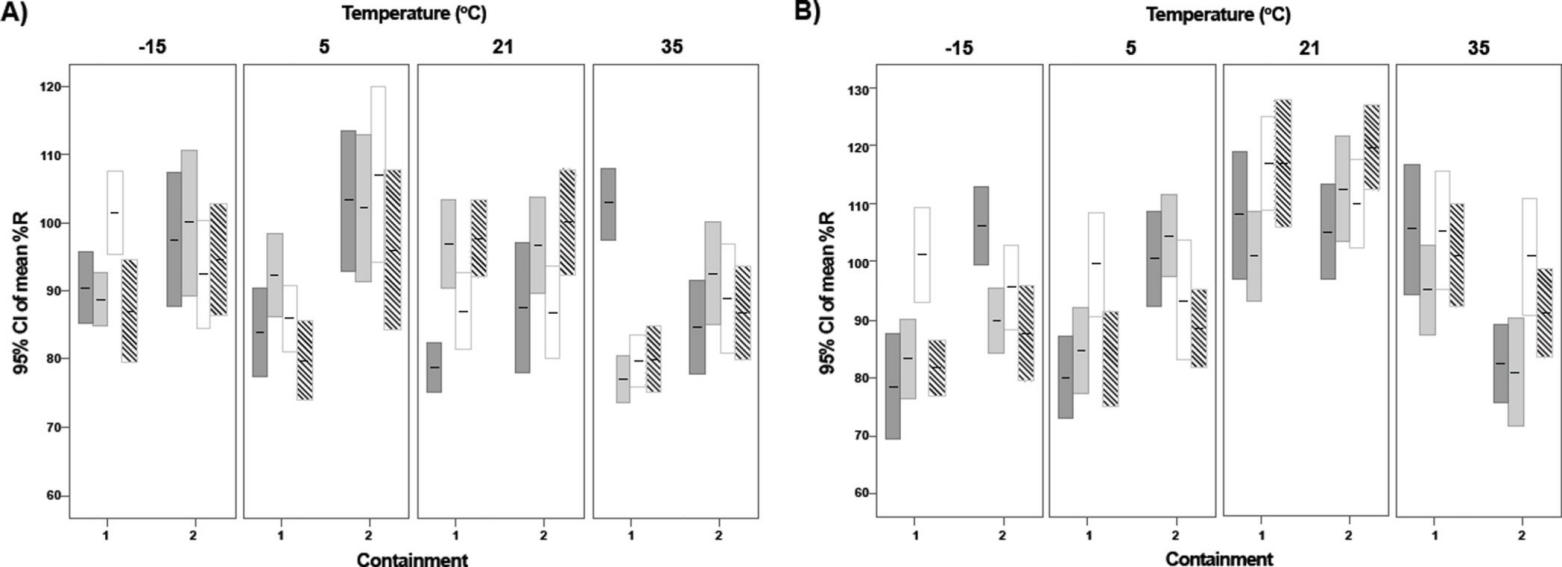
%R of BA from macrofoam swabs with dust and 104 spores and stored in (1) primary and (2) secondary containment. Bars represent 95% confidence intervals, n=120 for each containment, n=30 for each bar. Bars correspond with the following days: Day 1 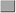, Day 2 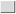, Day 4 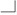, and Day 7 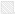. A: Inoculum 10^4^ CFU/swab, B: Inoculum 10^2^ CFU/swab.

The 95% confidence intervals of %R for each temperature and containment, relative to T_0_, at all sample points are illustrated in Figures 1A and B (10^4^ and 10^2^ CFU/swab). The %R pooled data for all swabs at both inoculum levels stored up to 2 days in SC, was significantly better (p ≤ 0.008) than if stored in PC. When swabs were stored at 5°C, the %R for both inoculum levels was significantly better if stored in SC, regardless of storage time (p ≤ 0.001, Figures 1A and B). The %R was significantly better than if in PC only (p ≤ 0.02) for swabs stored in SC at −15°C for up to 2 days, but no differences were seen between PC and SC if stored at 21°C for up to 7 days (p ≥ 0.23). If the 10^2^ data is pooled for up to 7 days, no significant differences are seen PC and SC (p ≥ 0.27), but at the higher 10^4^ inoculum level, SC provided a significantly better %R (p<0.001).

When the log_10_ transformed colony count data is examined, the greatest change for any storage variable tested was a 0.80 log_10_ decline which is seen when swabs were stored at 35°C for 7 days without ATD. The largest mean log_10_ change of swabs with ATD held at 5°C was −0.1 log_10_ seen at day 7 when held in PC and −0.05 log_10_ change when held in SC.

## Discussion

Maintaining organism viability during transportation and storage is always a concern when receiving samples of unknown concentrations. Previous research related to transportation and storage of organisms of clinical relevance has been conducted. *Streptococcus pneumoniae* was shown to maintain viability when stored at −70°C, but declined when the storage temperature was increased from 4°C to 30°C across a 4 day period [11]. *Neisseria gonorrhoeae* and *Streptococcus pneumoniae* experienced a decline of viability when samples were held at room temperature and processed within 48 hours of collection. The survival of organisms may be strain specific and not related to inoculum concentration [12]. Vegetative cells and spores of *Clostridium difficile* remained viable within a 56 day experiment, regardless of storage temperature (4°C/−20°C) and number of freeze, thaw, and refrigeration cycles [13]. Recommendations for most clinical specimens is to transport at 2–8°C or 25°C, depending on suspected organism, and to store in the laboratory at 4°C [21,22]. While packaging, requirements to protect personnel from select agents is specified in 49CFR173.196 [18], transportation and storage recommendations for maintaining sample integrity have not yet been established for BA spores. Samples collected from a bioterrorism event could potentially undergo temperature fluctuations due to non-optimal packaging, seasonal temperature fluctuations, high altitude air cargo conditions, or shipping delays. Typically, biological samples are shipped in insulated coolers with one or two cold packs to maintain temperatures at 2–8°C. How well these cold packs can maintain the cool temperatures under adverse conditions is uncertain. In one study, samples shipped with cold packs from Utah to Georgia over a 21 month period, temperature monitoring devices (I-button, Maxim Integrated Products, Inc., Sunnyvale, CA) noted fluctuations from −18° to +17°C (personal communication, C. Estill, CDC NIOSH, Cincinnati, OH).

Though BA spores are known to be heat tolerant [23] factors other than spore death may explain why some conditions are more favorable than others for recovery of spores from swabs. BA spores have a net negative charge and may adhere to the swab materials and release may be influenced by the spore coat interaction with the swab material, the pre-moistening agent, the presence of other dust and other organisms, and the storage temperature [24]. The current work sought to determine the best conditions for transporting swabs after sampling surfaces for BA spores. Sampling following decontamination carries an inherent risk of picking up residual disinfectant, which may cause false negative culture results because of disinfectant exposure during transit. NB was chosen as the preferred pre-moistening agent over PBST because it provided comparable recovery, it is commercially available, and is able to neutralize most commonly used disinfectants including chlorine containing disinfectants and quaternary ammonium compounds [25].

When comparing macrofoam swabs that were inoculated with and without ATD, %R from swabs with ATD was less variable between swabs, regardless of inoculum level. The ATD and other organisms present may interact electrochemically with the spores to reduce clumping in solution, allowing for more consistent CFU/ml counts between samples. In an actual sampling event, the composition of dust and background organisms will vary from site to site, and this effect may not hold true for all scenarios.

When the four temperatures were compared, storage at −15° and 5°C did not provide significantly different %R when data was pooled up to 2 days or up to 7 days, though maintaining a temperature of −15°C during shipping would involve the use of dry ice and more extensive packaging than if maintaining a temperature of 5°C.

Storage of swabs at 21°C or at 35°C provided less stability of the inoculum, with either significant increases or decreases in CFU/ml relative to T_0_. Storage at 35°C also encouraged growth of the background consortia (including other *Bacillus* spp.), making identification of the target BA colonies more difficult, especially at the 10^2^ inoculum density since the samples were not diluted before culture. However, a trained laboratorian was able to correctly identify the colonies with 100% and 96% sensitivity at the 10^4^ and 10^2^ inoculum levels, respectively, as confirmed by qPCR.

It should be noted that though fluctuations may appear large when presented as %R, the log_10_ transformed data revealed that the worst storage conditions (35°C for 7 days, PC, without ATD) resulted in ≤ 0.80 log_10_ decline in CFU/ml relative to T_0_. The best storage condition (5°C for 1 day, SC, with ATD) resulted in <0.01 log_10_ change in CFU/ ml relative to T_0_.

The data supports current CDC/NIOSH [14] recommended practices of shipping BA swab environmental samples at 5°C and processing within 48 hours of sampling, and provides additional data indicating SC provides better stability for the sample. Deviations from these practices may result in a difference in recovery up to 0.80 log_10_.

## Figures and Tables

**Table 1:** Mean %R and CV of swabs inoculated with 10^4^ and 10^2^ spores, with and without ATD, and stored at all temperatures in PC.

primer and probe set	Organic soil	Mean %R up to 2D*	CV	Mean %R up to 7D†	CV

10^4^	None	108.1%	0.66	105.0%	0.70
	With Dust	88.9%	0.18	88.1%‡	0.18

10^2^	None	86.8%	0.38	90.2%	0.44
	With Dust	92.0%	0.28	96.4%	0.27

* Data for 1 and 2 days pooled, n=240.

† Data for 1, 2, 4, and 7 days pooled, n=480.

‡ Three outliers omitted, n=477.

**Table 2:** Sensitivity of culture assay at each inoculum level (CFU swab^−1^) and storage temperature as confirmed by qPCR of selected BA colonies.

Inoculum (CFU/swab)	Temperature	Colonies confirmed positive	Sensitivity

10^4^	−15°C	50/50	100%
	5°C	50/50	100%
	21°C	50/50	100%
	35°C	50/50	100%

10^2^	−15°C	48/50	96%
	5°C	47/50	94%
	21°C	49/50	98%
	35°C	48/50	96%

**Table 3:** Mean %R and CV of macrofoam swabs inoculated with 10^4^ and 10^2^ spores, with ATD, and stored in SC.

Inoculum (CFU/swab)	Temperature	Mean %R up to 2D*	CV	Mean %R up to 7D†	CV
10^4^	−15°C	98.8%	0.28	96.1%	0.26
	5°C	102.7%	0.27	102.1%	0.30
	21C	92.2%	0.25	93.0%‡	0.23
	35°C	88.6%	0.22	88.2%	0.22
10^2^	−15°C	98.0%	0.19	94.8%	0.21
	5°C	102.5%	0.20	96.7%	0.23
	21°C	108.8%	0.21	111.8%	0.20
	35°C	81.7%	0.25	89.1%‡	0.26

* Data for 1 and 2 days pooled, n=60.

† Data for 1, 2, 4, and 7 days pooled, n=120.

‡ Three outliers omitted, n=117.

**Table 4:** P-values for comparison of swab storage at 5°C to −15, 21 and 35°C. Macrofoam swabs were inoculated with 10^4^ and 10^2^ spores, ATD, and stored in SC.

Inoculum (CFU/swab)	Storage temperatures as compared to storage at 5°C	P-value, up to 2D†	P-value, up to 7D‡

10^4^	−15°C	.817	.228
	21°C	.092	.021*§
	35°C	.000*	.010*

10^2^	−15°C	.639	.914
	21°C	.342	.000*
	35°C	.000*	.040*§

* P-value is significant.

† Data for 1 and 2 days pooled, n=60.

‡ Data for 1, 2, 4, and 7 days pooled, n=120.

§ Three outliers omitted, n=117.
